# What added value does Patient and Public Involvement (PPI) in oncology research bring to cancer patients and what are the challenges in realizing it? A mixed-methods cross-sectional study in four PPI groups in Flanders (Belgium)

**DOI:** 10.1186/s40900-026-00925-1

**Published:** 2026-07-01

**Authors:** Brent Taels, Florence Horicks, Silke Léonard, Teodora Lalova-Spinks, Ine Van Zeeland, Willy Dirkx, Lyzette Bax, Bernard Carton, Melissa De Regge

**Affiliations:** 1 Department of Marketing, Innovation and Organisation, Faculty of Economics and Business Administration, Ghent University, Ghent, Belgium; 2 LUCAS – Centre for Care Research and Consultancy, KU Leuven, Leuven, Belgium; 3 Department of Health Services Research, Care and Public Health Research Institute, Faculty of Health, Medicine and Life Sciences, Maastricht University, Maastricht, The Netherlands; 4 Institute for Interdisciplinary Innovation in Healthcare (I3h), Faculty of Medicine, Université Libre de Bruxelles, Brussels, Belgium; 5 Université Libre de Bruxelles, Pôle Santé, Brussels, Belgium; 6 Department of Health and Life Sciences, AP University of Applied Sciences and Arts, Antwerp, Belgium; 7 Department of Public Health and Primary Care, Faculty of Medicine and Health Sciences, Ghent University, Ghent, Belgium; 8 Centre for IT and IP Law, Faculty of Law, KU Leuven, Leuven, Belgium; 9 Clinical Pharmacology and Pharmacotherapy, Department of Pharmaceutical and Pharmacological Sciences, Faculty of Pharmaceutical Sciences, KU Leuven, Leuven, Belgium; 10 Symphony of Us project, Brussels, Belgium; 11 Ghent University Hospital, Strategic Policy Cell, Ghent, Belgium

**Keywords:** Patient and public involvement, PPI, Value, Oncology research, Cancer research

## Abstract

**Background:**

Despite group-based patient and public involvement (PPI) initiatives in oncology research being a growing trend, little research has been conducted on the added value of PPI according to the personal experiences of its contributors. This study aims to describe the personal experiences of PPI group members and coordinators in Flanders (Belgium) to investigate the dimensions of individual and collective added value of group-based PPI initiatives as well as the challenges in realizing this added value.

**Methods:**

All four PPI groups in Flanders relevant to oncology research were purposefully selected. The study used a mixed-method convergent parallel design. Quantitative and qualitative data were simultaneously collected using survey questionnaires and individual interviews – targeted at PPI group members, and one focus group – targeted at PPI group coordinators. Quantitative data were descriptively analyzed using univariate and bivariate analysis. Qualitative data were analyzed according to the Framework Method applying both deductive and inductive coding. Quantitative and qualitative data were integrated during interpretation of results using a side-by-side comparison.

**Results:**

A total of *N* = 52 PPI group members (RR: 46%) filled out the survey, out of which the responses of 43 members (38%) were retained for data analysis. *N* = 7 members who filled out the survey were subsequently individually interviewed, while *N* = 6 PPI coordinators participated in the focus group. PPI groups create individual added value for their group members as participation provides them with a sense of usefulness and belonging, while learning more about (oncology) research. Furthermore, PPI groups currently create collective added value by improving research feasibility. Potential collective added value creation lies in future roles of PPI groups such as guiding early design to enhance acceptability of evidence-based practices and supporting research dissemination to stimulate a democratization of knowledge.

**Conclusions:**

This study expands the value-based argument for PPI and connects it to the consequentialist argument. The study calls for future impact research to develop impact criteria that consider all relevant dimensions of collective added value creation as well as tackling persistent challenges that prevent added value creation.

**Supplementary Information:**

The online version contains supplementary material available at 10.1186/s40900-026-00925-1.

## Abstract (plain language summary)

Group-based patient and public involvement (PPI) initiatives – in which groups of patients such as patient advisory boards are consulted by researchers – are becoming important players in oncology research. However, few studies have addressed the question of what the added value of such initiatives are (and the challenges in realizing this added value) according to the personal experiences of its members and coordinators. This study therefore describes the personal experiences of group members and coordinators in four PPI groups in Flanders (Belgium). From these accounts, the study shows that PPI groups create added value for individual PPI group members in the sense that participation makes them feel useful, gives them a sense of belonging and provides them with more knowledge on (oncology) research. PPI groups also create collective added value for cancer patients in general as they can improve the feasibility of studies. However, to achieve more collective added value creation, PPI groups should also be able to initiate research topics (that closely align with actual patient needs) and to help the dissemination of research results. Furthermore, persistent challenges for collective added value creation need to be addressed such as ensuring a better social and cultural mix of PPI group members, implementing standardized procedures to which group members receive feedback from researchers, and providing better funding of PPI groups.

**Supplementary Information:**

The online version contains supplementary material available at 10.1186/s40900-026-00925-1.

## Background

Patient and Public Involvement (PPI) is a growing trend in oncology research and care [1]. It is defined by the British medical association as the ‘*active participation of citizens*,* users*,* carers and their representatives in the development of healthcare services and as partners in their own healthcare’* [2]. Applied to research, this active participation leads to *‘research being carried out ‘with’ or ‘by’ members of the public rather than ‘to’*,* ‘about’ or ‘for’ them’* [3]. In the oncology research field, traditionally dominated by researchers and medical professionals, PPI has the ambition to involve patients and members of the public as partners in research rather than simply as subjects of research [4, 5]. Involvement in research can occur individually, i.e. by consulting individual patient experts, but more commonly through group-based initiatives such as patient advisory boards that are consulted by researchers within specific organizational contexts [6].

Two main arguments for including PPI in oncology research can be distinguished. Firstly, the value-based argument is driven by moral and democratic values. As patients are directly affected by care and treatment processes informed by scientific evidence, it is increasingly being seen as ‘morally just’ to involve them in scientific research. Moreover, this allows patients to influence scientific agenda-setting leading to a certain degree of democratization of research [7, 8]. Secondly, the consequentialist argument relates more to outcomes of involving patients. Although assessing the impact of PPI has only recently become a topic of research, positive outcomes have been found regarding the design, process and delivery of research [9]. Greenhalgh and colleagues [7] attribute these positive outcomes to patients bringing in *‘a real world and lived experience perspective improving the efficiency and effectiveness of research’*.

Within the fast-growing PPI literature in oncology, there is often more attention for the consequentialist argument than for the value-based argument. However, measuring the impact of PPI is only meaningful if we know the dimensions for which PPI can create added value. A further elaboration of the value-based argument is therefore needed [5, 10]. To this end, it is essential to examine the personal experiences of PPI contributors to assess the added value of existing PPI initiatives in oncology [11]. PPI contributors consist of PPI group members – (former) patients or patient representatives, as well as coordinators – who manage the group administratively or scientifically and act as a bridge between group members and researchers. Added value can be understood both individually, i.e. for involved group members, and collectively, i.e. for other cancer patients who benefit from cancer research that has been informed by PPI [12]. An exploration of the dimensions of individual and collective added value of PPI in oncology research – as well the challenges in realizing it – can be an asset for further shaping impact measurements.

This study aims to describe the personal experiences of PPI contributors, i.e. PPI group members and PPI coordinators, in Flanders (Belgium) to investigate the dimensions of individual and collective added value of group-based PPI initiatives in oncology research – as well the challenges in realizing it. As Hoven and colleagues [12] suggest, added value is understood here both individually – as added value for PPI contributors themselves, and collectively – for cancer patients in general. Flanders is an ideal testcase to further explore the value-based argument for PPI given its highly developed oncology research system and strong patient advocacy that is however only integrated variably into the system. PPI in oncology research is a relatively new phenomenon in Flanders in which – at the time this study was conducted – four groups are integrated into existing organizational structures in hospitals or funding NGOs.

## Methods

### Design

This study used a mixed-method convergent parallel design [13]. Quantitative and qualitative data were simultaneously collected and analyzed. Quantitative data targeted at PPI group members – using closed-ended questions in survey questionnaires – were used to describe the main characteristics of the participants and their main experiences with involvement. Qualitative data targeted at PPI group members – using open-ended questions in survey questionnaires and personal interviews, and targeted at PPI coordinators – using a focus group – were used to structure results and gain more in-depth insight into dimensions of individual and collective added value of PPI. The integration of both methods allowed grasping the research problem in both breadth and depth by providing a more comprehensive understanding than each method could achieve independently. The research methodology and its reporting are in line with *the quality of mixed methods studies in health services research (GRAMMS)* guidelines [14], which can be found in SUPPLEMENTARY FILE 1.

### Involvement of patient researchers

This study was conducted in the scope of the *Symphony of Us* project, which aims to better understand and implement patient value in oncology research. Aligned with the principles of transdisciplinary research [15], this study was co-created between four academic researchers with diverse backgrounds (social sciences, biomedical sciences, nursing and law) and four patient researchers involved in the design, questionnaire development, interview and focus group conduction as well as results interpretation. One of the patient researchers also had prior experience with PPI. Details related to the co-creation process are reported in the GRIPP2 checklist [16], which can be found in SUPPLEMENTARY FILE 2.

### Participant recruitment

Coordinators of all four Flemish PPI groups relevant to oncology research were purposefully contacted to make a call for participation amongst their members (*N* = 114). These are (former) patients as well as patient representatives who are currently involved or have been involved in research projects, not as a research subject but as a contributor to the design, implementation, dissemination or evaluation of studies. Table 1 presents the characteristics of the four PPI groups. While the main purpose of the hospital PPI groups (group 1–3) is to assess whether the research process of future or ongoing research is sufficiently patient-centered, group 4 consists of two patient commissions co-determining which research proposals deserve project funding from a patient point of view. The groups assess ongoing or planned fundamental, translational, clinical as well as psychosocial cancer research.

**Table 1:** Characteristics of the four PPI groups

	Focus	Affiliation	Members	Date of origin
Group 1	oncology	hospital	N = 17	2021
Group 2	oncology	hospital	N = 22	2021
Group 3	multi-pathology	hospital	N = 19	2021
Group 4	oncology	NGO	N = 56	2016 and 2018*

*PPI group 4 consists of two patient commissions that were founded on different dates

### Data collection

Data were collected from May 2025 – August 2025 using a three-step protocol comprising a survey questionnaire, individual interviews, and one focus group (all in Dutch language). The survey questionnaire – targeted to PPI group members – consisted of 24 close-ended (including categorical and Likert scale questions) as well as 2 open-ended questions. Apart from socio-demographic information, the questionnaire first tackled general questions on involvement within the PPI groups. Furthermore, group members were asked how they are experiencing their involvement related to supporting infrastructures and resources for PPI, feedback procedures and perceived impact. Finally, the questionnaire asked about the extent to which patients think their involvement in PPI creates added value to themselves as well as to cancer patients in general. The translated full survey questionnaire can be found in SUPPLEMENTARY FILE 3. A link to fill out the survey, including the call for follow-up online interviews were sent out by the PPI coordinators. Two reminders were sent.

Individual interviews mainly focused on an in-depth discussion of group members’ motivation to participate in a PPI group, whether it creates added value to cancer patients, and – if affirmative – what that added value may be. The translated full interview guide for PPI group members can be found in SUPPLEMENTARY FILE 4. Individual interviews lasted approximately 30 min and were led by one researcher and – upon availability – co-led by a patient researcher.

The focus group – targeted to coordinators of the four PPI groups – aimed to examine personal experiences with PPI and the perceived added value of PPI from the perspective of the organizational managers of these initiatives. The full focus group guide for PPI group coordinators can also be found in SUPPLEMENTARY FILE 4. The focus group was co-moderated by one researcher and one patient researcher and lasted approximately 90 min.

### Data analysis

Quantitative and qualitative data were analyzed independently. Quantitative data were descriptively analyzed in SPSS© using univariate and bivariate analysis, the latter related to discrepancies in the Likert response patterns. Missing data were not considered for analysis. Furthermore, response patterns in which no data were collected for more than 50% of the total number of survey items were not retained for analysis. The interviews and focus group were audio-recorded, transcribed verbatim, and thematically analyzed using MAXQDA 24© (along with the answers to the open-ended questions in the survey). Qualitative data were analyzed according to the Framework Method by Richie and Spencer [17] applying both deductive and inductive coding. Deductive coding related to dimensions of individual and collective added value of PPI, for PPI contributors and cancer patients respectively. Codes that emerged inductively from the material were grouped in these categories using axial coding. The resulting coding tree can be consulted in SUPPLEMENTARY FILE 5.

Integration of quantitative and qualitative data happened during organizing and interpreting results. Although the codes from the qualitative research – representing dimensions of added value of PPI as well as its challenges – were the guideline for ordering the results, the quantitative results were compared side-by-side with these codes for assessing convergence and divergence.

## Results

### Participant characteristics

#### Quantitative part

In the quantitative part of the study, a total of 52 out of 114 PPI group members completed the survey (response rate [RR] = 46%). The responses of 43 members (38%) were retained for data analysis after checking for missing values. Table 2 presents the main characteristics of the PPI group members and modality of their involvement within a PPI group. Most members identified themselves as patient representatives (37.2%), former patients (23.3%) or patients (20.9%). 51.2% of the members were male, 46.4% were female. 92.7% of the members were at least 50 years old and 87.8% were highly educated having at least a bachelor’s degree.

Furthermore, relating to the modality of involvement in a PPI group, most group members (92.9%) report being in contact with their group at least every two to three months, in physical meetings (in 65.9% of cases) or through email and online communication (in 58.5% and 48.8% of cases respectively). PPI group members most frequently evaluate study protocols (in 88.4% of cases) and patient-facing documents such as informed consent forms, information letters and recruitment material for patients, i.e. flyers, posters, etc. (in 62.8% of cases). Few PPI group members indicated formulating ideas for new research (in 18.6% of cases), while almost no respondents were involved in later research stages such as data collection (in 4.7% of cases), data analysis & interpretation (in 2.3% of cases) and dissemination of results (in 2.3% of cases). Most PPI group members (93%) indicated to have never received any financial compensation for these activities.

#### Qualitative part

In the qualitative part of the study, *N* = 7 PPI group members who filled out the survey were subsequently interviewed. Four of these participants were female, three were male; four identified as former patients, two as patients, and one as a patient representative. Furthermore, the focus group included *N* = 6 participating coordinators representing all four PPI groups. All PPI coordinators were responsible for the practical organization of the PPI group and thus formed the main point of contact for PPI group members. Participating coordinators had a managerial or biomedical background.

### Narrative presentation of integrated results

The integration of the quantitative and qualitative results after a side-by-side comparison are narratively presented below. The integration applied primarily to the collective added value of PPI in oncology research and the associated challenges. In the further presentation, the notion ‘*PPI group members’* refers to both survey respondents and interviewees having a (former) patient or patient representative profile (quotes will be attributed to a code, e.g. SR1 referring to survey respondent 1 or I1 referring to interviewee 1). Furthermore, the notion ‘*PPI coordinators’* refers to the focus group participants (quotes will be attributed to a code, e.g. C1 referring to a coordinator from PPI group 1). All quotes are the authors’ own English translations of the original Dutch.

**Table 2:** PPI group members’ characteristics and modality of involvement in a PPI group (N=43)

Demographics	
Profile of the PPI group member	
Patient representative	37.2 % (N = 16)
Former patient	23.3 % (N = 10)
Patient	20.9 % (N = 9)
Other	18.6 % (N = 8)
Gender*	
Male	51.2 % (N = 21)
Female	46.4 % (N = 19)
Other	2.4 % (N = 1)
Age*	
31 – 40	7.3 % (N = 3)
41 – 50	0 % (N = 0)
51 – 65	39 % (N = 16)
65 +	53.7 % (N = 22)
Degree*	
PhD	2.4 % (N = 1)
Master	43.9 % (N = 18)
Bachelor	41.5 % (N = 17)
Secondary education	12.2 % (N = 7)
Modality of involvement in a PPI group	
Frequency of contact within PPI group	
Monthly	42.9 % (N = 18)
Every 2-3 months	50 % (N = 21)
Less than 3 times a year	7.1 % (N = 3)
Communication within PPI group**	
Physical	65.9 % (N = 27)
Email	58.5 % (N = 24)
Online	48.8 % (N = 20)
Phase of involvement in research projects**	
Formulation of ideas for new research	18.6 % (N = 8)
Evaluation of study protocols	88.4 % (N = 38)
Evaluation of patient-facing documents	62.8 % (N = 27)
Data collection	4.7 % (N = 2)
Data analysis & interpretation	2.3 % (N = 1)
Dissemination of results	2.3 % (N = 1)
Other	2.3 % (N = 1)
Financial compensation for PPI activities	
No fee	93 % (N = 40)
Monetary fee	7 % (N = 3)

* Variable contains system missing values

** Percentage measured as percentage of cases (multiple response analysis)

### Dimensions of individual added value of PPI in oncology research for PPI group members

This aspect of the analysis relied solely on qualitative data, which point to three dimensions of individual added value which PPI groups can create for their group members.

**A. Sense of usefulness**

The feeling of doing *‘useful’* or *‘meaningful’* activities that can make a difference to research projects was frequently described as valuable. One PPI group member particularly emphasized feeling useful by being a role model for other patients: *‘You need to explain to people [other patients] how important it [PPI] is*,* not only for yourself but also for those who will come after you (I1)’*. Furthermore, PPI group members indicated feeling useful in implementing their professional competencies within their PPI group. This was especially the case for group members that were retired at the time of their participation and used to be professionally active in a biomedical or pharmaceutical field. As one PPI group member explained: *‘I have been active in the pharmaceutical sector for a long period*,* so in the medical world … I had to read many studies and that is why I thought to inform myself [about PPI group activities] so I could make myself useful (I6)’.*

**B. Sense of belonging**

The importance of finding a sense of belonging within a group as a main motivation to be involved in research projects was emphasized by PPI group members as well as coordinators. The fact that the group consists of peers – predominantly fellow (former) patients – was described to be valuable as members often meet other members with the same clinical condition or type of cancer. Interviewed PPI group members, however, strongly emphasized that the general patient perspective must remain intact. As one interviewed PPI group member experienced: *‘I notice members want to tell their life stories in meetings. There are of course people that think they need to explain everything from their own point of view*,* while this is not the purpose [of a PPI group]. We need to look at it [research projects] from the point of view of being a general patient. […] When participating in a PPI group*,* you need to adopt this point of view*,* to not only look at your own situation (I3)’.* In practice, PPI group members who do not have or have not had cancer themselves often assess oncological research projects from this broader point of view, which was also described as valuable. *‘I am not a cancer patient myself. But I think my experience with cancer patients and the fact that I have a complex clinical background allows me to provide a meaningful contribution. It is up to me to correctly place the relevance of my input within the context of a cancer patient’s situation (SR47)’.*

Furthermore, coordinators emphasized that value creation through facilitating a sense of belonging through group dynamics should be further stimulated to attract new members. As one coordinator mentioned: *‘What we also notice is that it’s not all about working and reflecting*,* instead the group feeling is equally important to create a sense of satisfaction – we are talking about collective participation here. […] So*,* it’s very important to stimulate that [as a coordinator] (C4)’.*

**C. Getting to know more about (oncology) research**

By being involved in research, PPI group members get to know more about (oncology) research in a detailed way, which was described as valuable. In particular, retired PPI group members or members who are currently not working due to their disease mentioned the need for a ‘*cognitive pastime’*. Furthermore, patients emphasized the value of learning more about (oncology) research in general regardless of their own condition: ‘*The training and information is really why we are here. We feel it is positive for us to not only learn something about our own disease (I5)*’. One coordinator emphasized that especially knowledge acquisition on general preclinical cancer research creates value for PPI group members: *‘What they [PPI group members] often do not realize is the complexity of cancer. However*,* if you explain this to them*,* you get the impression that a certain respect for scientists is growing. […] I think the meaning [of PPI] for them relates to explaining preclinical research. Clinical research is of course more straightforward. But realizing why it [progress in research] is not developing in a fast way*,* this they only get when understanding the complexity of the biology of cancer (C1)’.*

### Dimensions of collective added value of PPI in oncology research for cancer patients in general

Qualitative data point to four dimensions of collective added value which PPI groups can create for cancer patients in general. Quantitative data, however, show that group members are not always involved in typical research phases that fall within these dimensions, while wanting to be more involved in the future. This discrepancy is evidenced by the Type I disconfirmation scores in Table 3 as the percentage of respondents that indicated currently not to be involved in a specific phase while wanting to be involved as a PPI group member in the future. These discrepancies are qualitatively discussed further below.

**Table 3:** Type I* disconfirmation scores between current and desired phases of involvement in research projects (N = 43)

Research phases	Type I disconfirmation score
(1) Formulation of ideas for new research	20.9 % (N = 9)
(2) Evaluation of study protocols	4.6 % (N = 2)
(3) Evaluation of patient-facing documents	9.3 % (N = 4)
(4) Data collection	4.6 % (N = 2)
(5) Data analysis	20.9 % (N = 9)
(6) Data interpretation	23.3 % (N = 10)
(7) Dissemination of results	23.3 % (N = 10)

* Type I disconfirmation score: phases of research projects in which PPI group members indicate currently not to be involved (Likert Score 1 – 2) while desiring to be involved in the future (Likert score 4 – 5)

**A. Giving direction to research projects**

Giving direction to research projects relates to typical research phases such as the formulation of ideas (for new research projects) and data analysis or interpretation (for ongoing projects). Quantitative data indicate that 20.9% of members who are currently not involved in formulating new ideas indicated they want to be more involved in the future. Furthermore, while almost no PPI group members (2.3%) in the survey indicated to be involved in the data analysis and interpretation phases, 20.9% and 23.3% of respondents respectively indicated desiring to be more involved in the future.

Qualitative data further show that formulating new ideas is perceived as the most important element to give direction to research projects. However, this argument was often limited to psychosocial and applied pharmacological research. As far as fundamental biomedical research is concerned, PPI group members commented they may be less well equipped – due to limited knowledge – to formulate new research ideas. Instead, they commented PPI groups tend to prioritize biomedical studies and guide the continuation of existing research lines within their organizations. As one patient interviewee argued: *‘I think it is hard to answer the question of what is necessary without having received any medical training. I will never be able to answer that question. But if they [researchers] would provide me with a projection of niche topics that they want to concentrate on and specialize in*,* then I think I could provide an answer (I4)’.*

Coordinators confirmed that formulating ideas for new research projects can be a potential practice of added value for PPI groups, but that this is not currently part of the remit of their groups. As one coordinator explained: ‘*In our group*,* it is not so obvious that patients come forward with ideas themselves. I’m proposing this every time*,* but it just doesn’t happen. […] In the five years the PPI group has existed*,* I have never had a patient coming forward with an idea (C3)’.* Furthermore, coordinators specifically emphasized education and long-term engagement of PPI members as prerequisites for PPI groups to formulate ideas for new research. Nevertheless, coordinators also limited this potential future practice of PPI groups to research projects related to quality in psychosocial and applied pharmacological oncology research. As one coordinator argued: *‘We should not give false hope to patients that they can be involved in formulating ideas for new research. We are*,* for example*,* researching cell communication. Everyone who has not received thorough biomedical scientific training cannot form an opinion about that topic or provide direction for this kind of research. There are of course studies about quality of life and clinical studies. Here I think patients can be involved early on of course (C1)’.*

**B. Increasing the feasibility of research projects**

In the qualitative analysis, PPI groups were often referred to as a ‘*link between patients and oncology researchers’* related to increasing the feasibility of research projects through the evaluation of study protocols and patient-facing documents. The quantitative data show accordingly that these are the phases in which PPI group members in this study are most involved as 88.4% and 62.8% of survey respondents indicated to evaluate study protocols and patient-facing documents respectively. Related to study protocols, PPI group members regularly assess whether the burden to participate in clinical studies – physically and mentally – as set out in study protocols is sufficiently low to facilitate participant recruitment. As one survey respondent argued: *‘Only patients know the full patient trajectory. By providing input*,* the impact for patients [participating in studies as subjects] can be minimized guaranteeing a better participation to studies (SR34)’*. Regarding patient-facing documents, PPI group members regularly assess lay-readability to prevent patients who initially decided to participate in a study from changing their mind. Although PPI members noted improvements in the language of contemporary patient-facing documents, they still see themselves as responsible for ensuring these materials are patient-friendly – sometimes by tweaking a few words, other times by advocating substantial language changes.

Qualitative data further indicate that these activities currently provide a clear collective added value as they can significantly improve participant recruitment and thus the feasibility of research projects. As one PPI group member explained: *‘It is of course the intention to recruit as many as possible people in a study*,* which is often a problem*,* right? We once received a video clip from an oncologist*,* and this was nice*,* to visualize what the study is about and what his intention is. For me everything was perfectly explained but for a general patient this was not the case. This is really something that those important scientific people do not always realize*,* that it may be very complicated for patients*,* related to their condition*,* related to their fears. Here PPI groups can be very useful (I2)’.*

**C. Increasing the acceptability of research projects**

Quantitative results show 92.1% of survey respondents indicated to believe PPI currently increases the ‘patient voice’ in oncology research, and 78.9% of respondents also indicated this would lead to more acceptable research from a patient point of view. In the qualitative analysis, PPI group members further emphasized it is an important task for PPI groups to assess whether it is *‘meaningful’*, *‘expedient’* or *‘acceptable’* to have patients participate in clinical studies. The applications in clinical trials must be of added value from a patient point of view and line up with general patient needs. ‘*Research for the sake of research’* was not seen as desirable by PPI group members. As one survey respondent argued: *‘A study of which the benefits are not clear … and that may possibly delay death rather than meaningfully improve quality of life can be called into question. This urges reflection from the side of researchers (SR39)’.* However, even when research projects are initially evaluated as relevant for patients, PPI group members indicated it is equally important to evaluate whether proposed possible side effects of clinical or pharmaceutical treatments are seen as acceptable from a broad patient point of view. *‘Researchers or doctors … do not always consider whether side effects are acceptable for patients. A mentality putting mere results [of treatments] first cannot be the way to go (SR11)’.*

Furthermore, PPI coordinators in the focus group recognized the unique perspective of PPI group members on the needs of cancer patients in general. As one PPI coordinator commented: ‘*In research*,* we often focus on the most ideal therapy in terms of effect*,* but we actually forget whether it is also the ideal therapy for the patient (C2B)’.* However, two PPI coordinators specifically emphasized that – for PPI groups to provide collective added value by increasing the acceptability of research projects – groups should ideally be involved before the start of the clinical phase. From the moment potential options for applying fundamental research become clear, PPI groups can assess whether the proposed application – which would be further developed and tested in clinical studies – is in line with overall cancer patient needs. As one coordinator argued: ‘*We think it is important to already involve the voices of patients in translational research. In this phase*,* the application is often already clear*,* and then it is even more important that patients are listened to at that stage*,* because if you then get feedback*,* for example*,* that those applications are not desired by patients and that they need to be looked at differently*,* then it is better to do that in that phase instead of already being in a clinical phase (C4)’.*

**D. Improving the dissemination of research results**.

Quantitative data indicate that 23.3% of PPI group members were not being involved in the dissemination of research results, while desiring to be involved in the future. In the qualitative analysis, PPI group members as well as PPI coordinators further emphasized PPI groups can have a potential collective added value in connecting researchers with patient organizations as important actors in disseminating research results. On the one hand, within the study protocols they are evaluating, PPI groups can stimulate researchers to include concrete dissemination plans setting out which patient organizations will be involved and in what ways. By stimulating contact with patient organizations in advance, the risk of tokenism is already reduced in study protocols. As one PPI group member argued: *‘The big challenge is to ensure that patient organizations are not an alibi or a sham (I1)’.* On the other hand, it was recognized that researchers are not always aware of relevant patient organizations in the field, while PPI groups were perceived as the ideal actor to guide researchers to these organizations based on their own networks. As one coordinator commented: *‘A comment we sometimes get from patients [within the PPI group] is that they themselves also have a very strong network and that they can sometimes be an intermediary to further communicate results*,* not only to patients themselves but to their wider network*,* and that can be interesting when results should be disseminated (C4)’.*

### Challenges in realizing the added value of PPI in oncology research

#### Challenge 1. A discussion on whether to train PPI group members

Quantitative results show that 62.8% of surveyed PPI group members did not receive any training to be involved in research projects within their PPI group. Members that indicated having received at least one training, were educated about general principles of preclinical and clinical oncology research, tools to evaluate the patient-centeredness of research protocols as well as the role of the respective organization (in which the PPI group is embedded) within the oncology research system. Nevertheless, 90.7% of PPI group members indicated that they felt fully competent to participate in the activities within their respective PPI group.

However, the qualitative data point toward a discussion on whether to train PPI group members or not, which was mentioned as a remaining challenge for PPI activities. In the interviews and the focus group two conflicting visions emerged in terms of whether to educate PPI group members or not. On the one hand, there were advocates for more training for PPI group members. Although experiential knowledge was perceived to be an asset in evaluating the patient-centeredness of research, training can help PPI group members to see things from a broader perspective and to ‘*step over their own condition*’. As one PPI group member explained: ‘*My idea is that you need a minimum of basic training. […] Suppose you need to read a phase I study*,* and you really don’t know anything about it – then you start to go off-topic in discussions (I2)’*. On the other hand, there were advocates to not *‘over-educate and professionalize’* PPI group members as this would lead to more scientific and methodological discussions and, as a result, a decrease of the aptitude of patients to bring up their perspectives. As one coordinator explained: *As you start educating patients more*,* they get into the way of experts and what we really want to avoid is that scientific discussions arise in the PPI group. […] We want patients to remain patients looking into patient-centeredness [of research] (C4)’.*

#### Challenge 2: The need for more diversity within PPI groups

Qualitative data indicate that the lack of diversity within contemporary PPI groups was mentioned as a substantial challenge for the future of PPI in oncology research. As quantitative data accordingly show, 92.7% of PPI group members in the survey were at least 50 years old, while 87.8% of members were highly educated having at least a bachelor’s degree. A minority of surveyed PPI group members (23.8%) indicated that their PPI group currently reflects social and cultural diversity within society.

In the qualitative analysis, PPI group members specifically mentioned the importance of having lower-educated members within the group in terms of increasing the feasibility of projects. As one member having a medical background explained: ‘*I find it interesting that we [the PPI group] have various profiles*,* it is always an advantage*,* because I do notice that I comment less on the lay-readability [of patient-facing documents] because I do understand the [medical] language used*,* but I notice that some patients say that there should be more clarity about the medical language (I6)’.* A surveyed PPI group member linked this situation to low health literacy skills: *‘For people with low health literacy*,* it is a big challenge to evaluate research. Yet*,* such people comprise a significant part of the patient population. As a PPI group member*,* my aim is to give them a voice* *(SR12)’*. Furthermore, one PPI coordinator mentioned that particularly psychosocial oncology projects would benefit from more social and cultural diversity within PPI groups. *‘While clinical treatment is the same for everyone*,* regardless of one’s background or education*,* psychosocial concerns are not the same. Psychosocial themes towards resuming work after treatment*,* fatigue*,* communication with children*,* these are all things that may be very different for various people within the target group (C4)’.*

Nevertheless, two limitations were mentioned regarding increasing the social and cultural diversity of PPI groups. First, reaching out to diverse groups of potential members leads to extra time and effort and, ultimately, also extra costs for coordinators to take up this task. As one coordinator experienced: ‘*Recently*,* we have been thinking thoroughly about how we can improve this*,* how we can set up PPI that does reach more diverse target groups. […] If you look at everything you would have to set up to reach a large target group*,* you need someone [a coordinator] to do this [job] full-time*,* which is just not feasible now (C1B)’.* Second, another coordinator emphasized that the start-up character of the PPI groups in Flanders benefits from the input of highly educated members to co-create and shape the initiatives. Reaching a more diverse group of PPI members can follow at the time initiatives have become more mature by using the networks of the members themselves. This was confirmed by one of the interviewed members: *‘Maybe we as former patients can approach those people [with more socially and culturally diverse backgrounds]*,* on a personal level*,* to take a small step forward (I4)’.* According to coordinators, an ideal situation consists of a well-balanced mix of profiles within a PPI group and a set of individual responsibilities tailored to members’ respective backgrounds and talents.

#### Challenge 3: The need for standardized feedback procedures

While in the quantitative analysis 76.7% of surveyed PPI group members indicated to generally feel appreciated by researchers, the qualitative analysis points to improving feedback procedures as an ongoing challenge in realizing the added value of PPI groups. Quantitative data show that 38.1% of the members indicated that they do not regularly receive feedback on their contributions to research projects, be it regarding providing ideas for new research or evaluating planned research and associated patient-facing documents. Moreover, qualitative data show that current feedback procedures are not fixed as feedback is sometimes provided and sometimes not.

Nevertheless, in the qualitative analysis feedback was described by PPI group members as well as coordinators as *‘interesting’* and *‘useful’*. Feedback can be provided directly by researchers in physical or online meetings or by laboratory field visits as well as indirectly through coordinators. Both PPI group members and coordinators emphasized the need for standardized feedback procedures relating to both intermediary and end results of research projects to which the PPI group was involved. Coordinators allocated themselves an assertive role in addressing researchers to provide feedback. ‘*It remains difficult*,* we try to send out an email that we are open to them*,* so that they can come and pitch their project to us. We try to send that out to as many researchers as possible*,* but it’s often the same ones that come back. We really must actively search and address them about this (C2A)’.*

#### Challenge 4: The need for more internal and external support for PPI groups

Especially in the qualitative analysis, the invisibility of PPI groups within their respective organizational structures was seen as a challenge to create added value. Increased visibility is necessary according to PPI group members as well as coordinators to attract a large and stable number of members. As one PPI group member explained: ‘*I didn’t know it [PPI group] existed before I saw the call. I assume that many – even if confronted with cancer themselves or within their families – don’t know that there is such a thing [as a PPI group]. But if you don’t know that*,* you may not feel compelled to participate (I3)’.*

Furthermore, the qualitative analysis emphasized the role of PPI coordinators in supporting and guiding PPI group members to achieve meaningful involvement. ‘*The guidance is also very important. To have someone who can guide patients from A to Z*,* making sure there are ample opportunities for contact*,* not just giving them a task to carry out (C4)’.* However, this supporting role was only seen as feasible by attracting sufficient internal and external funding. PPI in research remains a phenomenon that receives little financing and is also not always fully incorporated in internal hospital policies and financing. Furthermore, for most major research funding agencies or pharma companies, PPI is currently a non-binding activity for which no funding is provided. Coordinators in the focus group therefore highlighted the need for an umbrella organization for PPI in Flanders/Belgium, inspired by practices abroad, but noted that such an organization would entail substantial costs.

## Discussion

This mixed-methods cross-sectional study explores the individual and collective added value of group-based patient and public involvement (PPI) initiatives as a relatively new phenomenon in the oncology research system in Flanders (Belgium). The qualitative findings indicate three dimensions of individual added value which PPI groups can create for their group members, while integrated findings point to four dimensions of collective added value which PPI groups can create for cancer patients in general. By participating in a PPI group, members find a sense of usefulness and belonging and get to know more about (oncology) research. Collective added value is currently mainly realized in facilitating participant recruitment through evaluating study protocols and patient-facing documents and, subsequently, increasing the feasibility of research.

However, to create more collective added value for cancer patients in general, additional efforts by the oncology research system in Flanders regarding the implementation of PPI are necessary. As previously evidenced within the PPI literature [4, 18], this study similarly illustrates how PPI activities are concentrated at one specific point in the research trajectory: the moment in which research is already planned and about to start. PPI groups are mainly involved in evaluating study protocols and associated patient-facing documents that have already been set out by researchers. Although the groups certainly create collective added value in doing so, limiting the work package of the groups to these two activities can quickly lead to tokenism as – in contrast to the international definition of PPI – research is still not being carried out *‘with’ or ‘by’* its members [19, 20].

In Flanders as well as internationally, there is still a need to extend the activities of PPI groups to the entire research process of studies and to report these activities accordingly [21]. This study identifies two potential future roles for PPI groups that lead to the strengthening of evidence-based practices, on the one hand, and the democratization of scientific knowledge, on the other. The first future role relates to PPI groups helping to give direction to research in the early design phase – particularly in initiating relevant topics for psychosocial research as well as biomedical research through evaluating whether applications in translational and clinical studies align with general cancer patient needs. PPI groups can thus increase the acceptability of research, which could significantly reduce the chances of producing ‘irrelevant and wasteful’ studies [22, 23]. This role would maximize the chances that oncological care and treatment processes resulting from the research bring into practice what patients value, clearly making resulting evidence-based practices more acceptable to patients. This includes a care and treatment trajectory that keeps patients in control (through therapeutic alliances, shared-decision making and self-care practices) and that prepares for and addresses survivors’ physical sequelae as well as their psychological and social challenges [24]. A second future role relates to PPI groups facilitating the dissemination of research findings by stimulating researchers to adopt concrete dissemination plans as well as guiding researchers to relevant patient organizations. As a result, this role would significantly facilitate the spreading of research results beyond the scientific and academic sphere stimulating a democratization of knowledge [4], which can only benefit cancer patients in general.

The findings of this study extend prior work in the PPI literature by expanding the value-based argument for PPI and directly connecting it to the consequentialist argument. It is not only morally just to involve patients – as end-users of evidence-based care – in the research projects that form the foundation of that care. Instead, patients in this study predominantly describe their participation in a PPI group as valuable for cancer patients in general. Hence, the value-based argument directly intersects with the consequentialist argument for PPI. As research increasingly focuses on the impact of PPI [25], future research agendas are encouraged to develop impact criteria that go beyond recruitment but also take any other potential contributions of PPI groups in creating collective added value into account. Impact measurements could therefore also examine the ability for PPI groups to initiate research topics, the extent to which PPI groups align research topics more closely with the actual needs of patients, as well as the extent to which PPI groups are involved in the dissemination strategy at the end of the research process. Future critical research should investigate whether and how PPI influences the relationships between patients, members of the public and researchers, and how it affects research more broadly [26].

Nevertheless, this study confirms several challenges particularly for collective added value creation previously described within the PPI literature. First and foremost, PPI group structures can be enhanced by ensuring a better mix of group members regarding social, educational and cultural backgrounds. Although participants indicated that particularly involving group members with a lower level of education poses a challenge, the current degree of homogeneity within PPI groups was viewed as a potentially threatening factor to collective added value creation. Too much homogeneity can lead to blind spots in, for example, weighing the needs of diverse patient groups or evaluating the patient-friendliness of patient-facing documents [27, 28]. Furthermore, this study highlights the need for implementing standardized feedback procedures and the important role of PPI coordinators herein [11]. PPI group members are volunteers who often participate in PPI activities free of charge, making feedback on their work not only necessary for developing collective experiential knowledge and expertise within the groups, but also an ethical imperative. Standardized feedback may lead to further reflection on the part of researchers and, hence, a better incorporation of the patient’s perspective within planned research. Finally, it shows the need for better funding of PPI groups [29], which may increase the diversity of their members and provide coordinators with more opportunities for guidance and support. A form of actual financial compensation for group members may also help to keep them on board of the PPI group on a structural basis. The preceding aspects would all enhance the further development and collective learning process of PPI groups which would ultimately benefit all cancer patients.

This study is – to our knowledge – the first to explore PPI practices in oncology research in a Belgian context as well as one of the first to include the experiences of (former) patients and patient representatives as the main contributors to PPI. A first strength of this study is that patient researchers – one of whom also had prior experience in PPI group activities – co-designed and co-conducted the study. A second strength of this study is that it answers a call in the literature to further explore the value-based argument for PPI [10] by particularly including the experiences of PPI contributors [11]. However, the study has one main limitation: the absence of contrasts in the analysis of experiences of PPI group members and coordinators. While our aim was to describe added value creation of PPI groups according to the experiences of both profiles, future studies would benefit from analytically exploring any differences in attitudes, perceptions and experiences. The inclusion of researchers – some of whom approach PPI groups on their own initiative while others are forced to do so by funding agencies – is also an important point in this regard.

## Conclusion

Although group-based patient and public involvement (PPI) initiatives are a relatively new phenomenon in Flanders (Belgium), it provides individual added value for group members and has clear potential for collective added value creation for cancer patients in general. PPI groups currently provide collective added value by improving research feasibility through reviewing study protocols and patient-facing documents of planned research. However, this study identifies two potential future roles by which PPI groups could create more collective added value: giving direction to research in the early design phase to make evidence-based practices more acceptable to patients, and facilitating the dissemination of research findings to stimulate a democratization of knowledge. This study calls for an expansion of the value-based argument for PPI and connects it to the consequentialist argument. Future impact research should develop impact criteria that consider all relevant dimensions of collective added value creation. These include the ability of PPI groups to initiate research topics, the extent to which they align research topics with actual patient needs, and the extent to which the groups are involved in the dissemination strategy. Furthermore, persistent challenges that prevent added value creation should be tackled by ensuring a better social and cultural mix of group members, implementing standardized feedback procedures, and providing better funding of PPI groups.

## Supplementary Information

Below is the link to the electronic supplementary material.

Supplementary Material 1

Supplementary Material 2

Supplementary Material 3

Supplementary Material 4

Supplementary Material 5

## Data Availability

The datasets generated and/or analyzed during the current study are not publicly available due to legal and ethical constraints. Study participants did not provide consent for the sharing of their data with parties other than the researchers.

## References

[1] Farah E, Kenney M, Kica A, Haddad P, Stewart DJ, Bradford JP. Beyond Participation: Evaluating the Role of Patients in Designing Oncology Clinical Trials. Curr Oncol. 2023;30(9):8310–27. 37754518 10.3390/curroncol30090603 PMC10527717

[2] British Medical Association. Patient and public involvement: a tool kit for GPs. London; 2015.

[3] National Institute for Health and Care Research. Public involvement in research. Available from: https://www.nihr.ac.uk/get-involved/public-involvement.

[4] Colomer-Lahiguera S, Steimer M, Ellis U, Eicher M, Tompson M, Corbière T, Haase KR. Patient and public involvement in cancer research: A scoping review. Cancer Med-Us. 2023;12(14):15530–43. 10.1002/cam4.6200 PMC10417078 37329180

[5] Shakhnenko I, Husson O, Chuter D, van der Graaf W. Elements of successful patient involvement in clinical cancer trials: a review of the literature. Esmo Open. 2024;9(4). 10.1016/j.esmoop.2024.102947 PMC10959641 38492274

[6] Price A, Clarke M, Staniszewska S, Chu L, Tembo D, Kirkpatrick M, Nelken Y. Patient and Public Involvement in research: A journey to co-production. Patient Educ Couns. 2022;105(4):1041–7. 34334264 10.1016/j.pec.2021.07.021

[7] Greenhalgh T, Hinton L, Finlay T, Macfarlane A, Fahy N, Clyde B, Chant A. Frameworks for supporting patient and public involvement in research: Systematic review and co-design pilot. Health Expect. 2019;22(4):785–801. 31012259 10.1111/hex.12888 PMC6737756

[8] Pii KH, Schou LH, Piil K, Jarden M. Current trends in patient and public involvement in cancer research: A systematic review. Health Expect. 2019;22(1):3–20. 30378234 10.1111/hex.12841 PMC6351419

[9] Modigh A, Sampaio F, Moberg L, Fredriksson M. The impact of patient and public involvement in health research versus healthcare: A scoping review of reviews. Health Policy. 2021;125(9):1208–21. 34376328 10.1016/j.healthpol.2021.07.008

[10] Laidlaw L, Hollick RJ. Values and value in patient and public involvement: moving beyond methods. Future Healthc J. 2022;9(3):238–42. 36561805 10.7861/fhj.2022-0108 PMC9761452

[11] Mathie E, Smeeton N, Munday D, Rhodes G, Wythe H, Jones J. The role of patient and public involvement leads in facilitating feedback: invisible work. Res Involv Engagem. 2020;6:40. 32676199 10.1186/s40900-020-00209-2 PMC7353750

[12] Hoven E, Eriksson L, Mansson D, Souza A, Sorensen J, Hill D, Viklund C, et al. What makes it work? Exploring experiences of patient research partners and researchers involved in a long-term co-creative research collaboration. Res Involv Engagem. 2020;6:33. 32579132 10.1186/s40900-020-00207-4 PMC7305606

[13] Creswell JW, Clark VLP. Designing and conducting mixed-methods research (Third edition) Sage Publications. 2017.

[14] O’Cathain A, Murphy E, Nicholl J. The quality of mixed methods studies in health services research. J Health Serv Res Po. 2008;13(2):92–8. 10.1258/jhsrp.2007.007074 18416914

[15] Pohl C, Krütli P, Stauffacher M. Ten Reflective Steps for Rendering Research Societally Relevant. Gaia. 2017;26(1):43–51.

[16] Staniszewska S, Brett J, Simera I, Seers K, Mockford C, Goodlad S, et al. GRIPP2 reporting checklists: tools to improve reporting of patient and public involvement in research. Bmj-Brit Med J. 2017;358. 10.1136/bmj.j3453 PMC5539518 28768629

[17] Ritchie J, Spencer L. Qualitative data analysis for applied policy research In: Bryman A, & Burgess, R., editor. Analyzing Qualitative Data London Routledge 1994.

[18] Selman LE, Clement C, Douglas M, Douglas K, Taylor J, Metcalfe C, et al. Patient and public involvement in randomised clinical trials: a mixed-methods study of a clinical trials unit to identify good practice, barriers and facilitators. Trials. 2021;22(1):735. 34688304 10.1186/s13063-021-05701-y PMC8542312

[19] Jackson T, Pinnock H, Liew SM, Horne E, Ehrlich E, Fulton O, et al. Patient and public involvement in research: from tokenistic box ticking to valued team members. Bmc Med. 2020;18(1). 10.1186/s12916-020-01544-7 PMC7153227 32279658

[20] Schilling I, Gerhardus A. Is this really Empowerment? Enhancing our understanding of empowerment in patient and public involvement within clinical research. BMC Med Res Methodol. 2024;24(1):205. 39272031 10.1186/s12874-024-02323-1 PMC11395860

[21] Vanneste A, Wens I, Sinnaeve P, Louati C, Huys I, Ioannidis JPA, Adriaenssens T. Evolution of reported patient and public involvement over time in randomised controlled trials in major medical journals and in their protocols: meta-epidemiological evaluation. BMJ (Clinical Res ed). 2025;389:e082697. 10.1136/bmj-2024-082697 PMC11983158 40210257

[22] Engelaar M, Bos N, van Schelven F, Lorenzo ISN, Couespel N, Apolone G, et al. Collaborating with cancer patients and informal caregivers in a European study on quality of life: protocol to embed patient and public involvement within the EUonQoL project. Res Involv Engagem. 2024;10(1):59. 38863075 10.1186/s40900-024-00597-9 PMC11167745

[23] Piil K, Johannessen KB, Pappot H. Strategies for meaningful patient and public involvement in neuro-oncological research. Neuro-oncology Pract. 2024;11(2):109–10. 10.1093/nop/npad080 PMC10940832 38496919

[24] Horicks F, Lalova-Spinks T, Léonard S, Remy D, Taels B, Stevens H. Self-reported patient experiences in a peer-support community: What do cancer patients value? Front Psychol. 2026;17(1724101). 10.3389/fpsyg.2026.1724101 PMC13003601 41867993

[25] Fredriksson M, Sampaio F, Moberg L. The impact of patient and public involvement in healthcare services: a conceptual review spanning social sciences and health sciences. Ssm-Qual Res Health. 2025;7.

[26] Russell J, Fudge N, Greenhalgh T. The impact of public involvement in health research: what are we measuring? Why are we measuring it? Should we stop measuring it? Res Involv Engagem. 2020;6:63. 33133636 10.1186/s40900-020-00239-w PMC7592364

[27] Gathani T, Dhaliwal B, Lwiindi J, Dawson S. Lessons learnt from developing an ethnically diverse patient and public involvement group for breast cancer research. Bmj Open. 2025;15(3). 10.1136/bmjopen-2024-091888 PMC11877151 40032390

[28] Green S, Santaolalla A, Russell B, George G, Wylie H, Monroy-Iglesias M, et al. Addressing health disparities and equitable representation in cancer research through the initiative, ‘The Patient and Public Involvement Cancer Research Group for Diverse Backgrounds’. Ecancermedicalscienc. 2025;19. 10.3332/ecancer.2025.1885 PMC12149224 40496318

[29] Ganz-Blaettler U, Liptrott SJ, Tolotti A, Cefali M, Aeschlimann C, Vilei SB, et al. The active involvement of patients in oncology research. Cancer Treat Rev. 2024;130. 10.1016/j.ctrv.2024.102822 39276429

