# Supplementary material for: What added value does Patient and Public Involvement (PPI) in oncology research bring to cancer patients and what are the challenges in realizing it? A mixed-methods cross-sectional study in four PPI groups in Flanders (Belgium)
Source: Res Involv Engagem. 2026 Jul 1;12:105. doi: 10.1186/s40900-026-00925-1 (PMC13326187; doi:10.1186/s40900-026-00925-1)
Supplement: Supplementary file 1 — Supplementary Material 1 [file 40900_2026_925_MOESM1_ESM.docx]

**Supplementary file 1. Good Reporting of A Mixed Methods Study (GRAMMS)**

**For the manuscript: What added value does Patient and Public Involvement (PPI) in oncology research bring to cancer patients and what are the challenges in realizing it? A mixed-methods cross-sectional study in four PPI groups in Flanders (Belgium)**

| **Guideline** | **Section** |
| --- | --- |
| **Describe the justification for using a mixed methods approach to the research question**  With this study our primary aim was to have more insights on the added value of PPI in oncology research in Flanders (Belgium) as well as existing challenges to realize it – according to the experiences of its contributors.  We used quantitative data to describe the main characteristics of the participants and the experiences of involvement, while qualitative data were used to were used to structure results and gain more in-depth insight into dimensions of individual and collective added value of PPI.  The integration of both methods allows grasping the research problem in both breadth and depth by providing a more comprehensive understanding than each method could achieve independently. | Method section:  -design |
| **Describe the design in terms of the purpose, priority and sequence of methods**  This study used a mixed-methods convergent parallel design, in which quantitative and qualitative data were simultaneously collected and analyzed. Qualitative data were used to structure the results, which would later be integrated with the quantitative results using a side-by-side comparison to allow the examination of convergence and divergence. | Method section:  -design  -data analysis |
| **Describe each method in terms of sampling, data collection and analysis**  Quantitative methods included purposefully sending out a survey questionnaire to N = 114 PPI group members in four group-based PPI initiatives in Flanders. The survey consisted of 24 close-ended and 2 open questions and included a call for follow-up interviews. Data analysis included a descriptive analysis in SPSS© using univariate and bivariate analysis.  Qualitative methods included individual interviewing with PPI members (who also completed the survey) as well as organizing a focus group with PPI coordinators representing the four purposefully selected group-based PPI initiatives in Flanders. Questionnaires were developed between the research team and patient researchers in co-creation as further explained in supplementary file 2. Qualitative data were inductively and deductively analyzed using the Framework Method. | Method section:  -participant recruitment  -data collection  -data analysis |
| **Describe where integration has occurred, how it has occurred and who has participated in it**  Integration of quantitative and qualitative data happened during organizing and interpreting results. Although the codes from the qualitative research – representing dimensions of added value of PPI as well as its challenges – were the guideline for ordering the results, the quantitative results were compared side-by-side with these codes for assessing convergence and divergence.  The integration of quantitative and qualitative data was discussed between the members of the research teams and the patient researchers. | Method section  -data analysis |
| **Describe any limitation of one method associated with the present of the other method**  The survey questionnaire was primarily designed to have descriptive information on the main characteristics of PPI group members as well as the main characteristics of their involvement within a PPI group. Although the questionnaire included open and closed-ended questions that tackled added value of PPI (and challenges) the qualitative methods were primarily designed to provide more in-depth information that could be further used in structuring the results. Nevertheless, the open-ended questions on value in the questionnaire still provided opportunities for participants who could not be interviewed to give their opinion on this subject. | Not specifically discussed within the manuscript itself |
| **Describe any insights gained from mixing or integrating methods**  We presented the integrated findings in response to the research problem narratively. We identified three dimensions of individual added value of PPI groups for which only qualitative data were applicable. Integrated findings primarily applied to collective added value of PPI groups, by which we identified four dimensions, as well as four persistent challenges in realizing this added value. A side-by-side comparison showed mainly convergence between quantitative and qualitative data, but also divergence in some cases (e.g. challenge 1). | Results section  -narrative presentation of integrated results  Discussion section |

*O'Cathain A, Murphy E, Nicholl J. The quality of mixed methods studies in health services research. J Health Serv Res Policy. 2008;13(2):92-98.*
