# Supplementary material for: What added value does Patient and Public Involvement (PPI) in oncology research bring to cancer patients and what are the challenges in realizing it? A mixed-methods cross-sectional study in four PPI groups in Flanders (Belgium)
Source: Res Involv Engagem. 2026 Jul 1;12:105. doi: 10.1186/s40900-026-00925-1 (PMC13326187; doi:10.1186/s40900-026-00925-1)
Supplement: Supplementary file 2 — Supplementary Material 2 [file 40900_2026_925_MOESM2_ESM.docx]

**Supplementary file 2. Reporting patient and public involvement in health and social care research (GRIPP2)**

**For the manuscript: What added value does Patient and Public Involvement (PPI) in oncology research bring to cancer patients and what are the challenges in realizing it? A mixed-methods cross-sectional study in four PPI groups in Flanders (Belgium)**

| **Section** | **Description of section** | **Answers** |
| --- | --- | --- |
| **Aim** | Report the aim of PPI in the study | Patients were involved to contribute to all phases of the study (study design, questionnaire development, interview and focus group conduction as well as results interpretation) with the principal aim of incorporating the patient perspective. The involvement of patients is mentioned throughout the manuscript as ‘patient researchers’. Collaboration with patient researchers was structurally embedded within the *Symphony of Us project*, of which this study was a part. |
| **Methods** | Provide a clear description of the methods used for PPIE in the study | Related to the study design, patient researchers were invited to co-create the survey questionnaires, i.e. to provide themes essential from a patient perspective in relation to existing literature, and to provide feedback on draft questionnaires set up by the researchers.  Related to the data collection, patient researchers were invited to participate in the interview and focus group conduction as co-researcher upon availability. Their participation was preceded by a preparatory meeting with the researcher leading this study (BT) before their participation in an interview or focus group. They also had the availability for a debriefing (via mail, telephone or online) after their participation.  Related to results interpretation, patient researchers were invited in a meeting with the researchers to discuss the results and to co-decide on interesting elements for the discussion within the manuscript. |
| **Study results** | Outcomes – Report the results of PPI in the study, including both positive and negative outcomes | A total number of four patient-researchers (structurally embedded within the *Symphony of Us project*) were involved voluntarily throughout this study upon availability (IVZ, WD, LB, BC).  Related to the study design, all four patient researchers presented ideas for the questionnaires in a physical meeting after a thorough presentation of existing literature on the topic by the researchers. Two patient researchers (IVZ and WD) were later involved in rephrasing, deleting or adding questions to the questionnaires developed by the researchers (BT, FH, SL, TLS).  Related to the data collection, two patient researchers (IVZ and WD) participated in one or more of the interviews as a co-researcher and one patient researcher (WD) participated as a co-researcher in the focus group.  Related to results interpretation, all four patient researchers shared their ideas on the results in a physical meeting. |
| **Discussion and conclusions** | Outcomes – Comment on the extent to which PPI influenced the study overall. Describe positive and negative effects | The involvement of patient researchers had multiple positive effects. Patient researchers first and foremost influenced the study through improving the questionnaire development, ensuring its relevance to PPI group members. Furthermore, their presence in some of the interviews and the focus group imposed critical reflections by interviewees and the PPI coordinators. Finally, their comments during debriefings and data analysis discussions led to critical reflections by the research team (and in some cases, a reinterpretation of the findings). Two patient researchers (IVZ and LB) also influenced the presentation of the findings within the manuscript by providing critical comments.  However, a challenge reported was the uneven and limited engagement from some of the involved patient researchers due to several reasons such as the voluntary character of their role and physical conditions. Furthermore, at times the research team experienced challenges in properly involving and preparing the patient researchers due to time constraints. |
| **Reflections/critical perspective** | Comment critically on the study, reflecting on the things that went well and those that didn’t, so others can learn from this experience | The collaboration with the patient researchers – despite the above-mentioned challenges – was productive, partially since they were already involved in the broader project *Symphony of Us*. Patient researchers described their involvement as meaningful. Preparing patient researchers (e.g. by providing them with a thorough oversight of the literature and methods on questionnaire development) and keeping them up to date of the study progress significantly helped to keep them on board. |

*Staniszewska, S., Brett, J., Simera, I., Seers, K., Mockford, C., Goodlad, S., et al. (2017). GRIPP2 reporting checklists: tools to improve reporting of patient and public involvement in research. bmj, 358.*
