# Supplementary material for: What added value does Patient and Public Involvement (PPI) in oncology research bring to cancer patients and what are the challenges in realizing it? A mixed-methods cross-sectional study in four PPI groups in Flanders (Belgium)
Source: Res Involv Engagem. 2026 Jul 1;12:105. doi: 10.1186/s40900-026-00925-1 (PMC13326187; doi:10.1186/s40900-026-00925-1)
Supplement: Supplementary file 3 — Supplementary Material 3 [file 40900_2026_925_MOESM3_ESM.docx]

**Supplementary file 3. Survey questionnaire**

**Q. Intro1:** By proceeding, you agree that your data are collected and processed for the purposes of this study.

- I agree to participate in this study and declare that my data may be collected and processed
- I do not want to participate in this study (SKIP LOGIC: end of survey)

**Q. Intro2:** How would you best identify yourself?

- Former patient
- Patient
- Patient representative
- Other patient profile (TEXT BOX: Please specify)

| ***Part 1. General questions on patient involvement in oncology research*** |
| --- |
| **The first part of the questionnaire contains a number of general questions on your involvement in oncological research projects.** |
| **Q1A.** Through which type of organization(s) did you come into contact with oncological research projects? (M)   - Hospital - Public research institution - Patient association - Pharmaceutical company - Ethical committee - Other (please indicate) |
| **Q1B.** Have you received training to contribute to research projects within any of the organizations you indicated in the previous question?   - Yes (TEXT BOX: What kind of training? And what were they about?) - No |
| **Q1C.** Is there any compensation from any of these organizations for the contribution you make to research projects? (M)   - Yes, a monetary amount - Yes, the legally established volunteer contribution in Belgium - Yes, material benefits such as gifts, vouchers, etc. - No |
| **Q1D.** How do you usually communicate with researchers or other responsible persons for patient involvement within any of these organizations? (M)   - Physical meetings - Online meetings - Email communication |
| **Q1E.** How often are you contacted by researchers or other responsible persons for patient involvement within any of these organizations?   - Every month - Every 2 to 3 months - Less than 3 times a year - I usually have to initiate contacts myself |
| **Q1F.** In what phase(s) of research projects have you been involved so far? (M)   - Formulating ideas for new research and/or drawing up new research proposals - Evaluating study protocols or research proposals - Evaluating patient-facing documents, i.e. informed consents and information letters - Collecting research data - Analyzing collected research data - Interpreting the analyzed data - Disseminating research results - Other (please indicate) |
| **Q1G.** In what phase(s) of the research – that you were not involved in so far – would you like to be more involved in in the future?   - Formulating ideas for new research and/or drawing up new research proposals - Evaluating study protocols or research proposals - Evaluating patient-facing documents, i.e. informed consents and information letters - Collecting research data - Analyzing collected research data - Interpreting the analyzed data - Disseminating research results - Other (please indicate) |
| ***Part 2. PPI and patient value in oncology research*** |
| **The second part questions the extent to which you think your involvement in research projects brings value to yourself as well as to cancer patients in general.** |
| **Q2A.** Based on my experiences, I do consider my involvement in research projects as meaningful for myself.   - Yes (Please indicate why.) - No (Please indicate why not.) |
| **Q2B.** Based on my experiences, I think my involvement in research projects is meaningful for other cancer patients.   - Yes (Please indicate why.) - No (Please indicate why not.) |
| **Q2C.** I feel valued by the professionals (e.g. care professionals and researchers) I work with.   - LIKERT SCALE |
| **Q2D.** I think that the patients who are currently involved in research projects are a good reflection (for example in social and cultural terms) of cancer patients in society as a whole.   - LIKERT SCALE |
| ***Part 3***. ***General experiences with patient involvement in oncology research*** |
| **The third part questions your general experiences with being involved in oncology research. Please indicate to what extent you agree with the statements below.** |
| **Q3A.** I know what is expected from me when I am involved in research projects.   - LIKERT SCALE |
| **Q3B.** It is clear to me what they want to achieve with patient involvement in research projects.   - LIKERT SCALE |
| **Q3C.** I find myself useful when I am involved in research projects.   - LIKERT SCALE |
| **Q3D.** I feel like I am being listened to as a patient (representative) when I am involved in research projects.   - LIKERT SCALE |
| **Q3E.** I believe that the way in which I am currently involved in research projects is sufficient to make a difference.   - LIKERT SCALE |
| ***Part 4. Experiences on supporting infrastructures for PPI*** |
| **The fourth part questions your experiences on the guidance and support you receive when being involved in oncology research. Please indicate to what extent you agree with the statements below.** |
| **Q4A**. I feel fully equipped to be able to contribute to research projects.   - LIKERT SCALE |
| **Q4B.** Within the patient involvement initiatives in which I am involved, there is a need for additional guidance and support for patients to contribute to research projects.   - LIKERT SCALE |
| **Q4C.** When I am involved in research, I am provided with sufficient time to contribute to research projects.   - LIKERT SCALE |
| **Q4D.** More resources and external support for meaningfully involving patients in research projects are needed.   - LIKERT SCALE |
| ***Part 5. Perceived impact of PPI*** |
| **This final part questions the extent to which you think you have had an impact on the research projects in which you have been involved.** |
| **Q5A.** I regularly receive feedback from researchers to know what difference I am making with my contributions to research projects.   - LIKERT SCALE |
| **Q5B.** I think my contributions have a clear impact on research projects.   - Yes (Display logic to Q5C.) - No |
| **Q5C.** Please complete. My contribution to research projects will likely increase …? (M)   - the patients’ voice in research - the relevance of research - the chances that research will meet the actual needs of cancer patients *(acceptability)* - the chances that patients can easily participate in research *(feasibility)* - the chances that research results closely match reality *(validity)* - the chances of a wider dissemination of research results |
| ***Part 6. Socio-Demographics*** |
| **Finally, we would like to ask you about some personal characteristics before ending the questionnaire.** |
| **Q6A.** What is your gender?   - Male - Female - Other |
| **Q6B.** Which age category are you in?   - 18 – 30 - 31 – 40 - 41 – 50 - 51 – 65 - + 65 |
| **Q6C.** What is your highest degree?   - PhD - Masters’ degree - Bachelors’ degree - Secondary education |
