# Supplementary material for: What added value does Patient and Public Involvement (PPI) in oncology research bring to cancer patients and what are the challenges in realizing it? A mixed-methods cross-sectional study in four PPI groups in Flanders (Belgium)
Source: Res Involv Engagem. 2026 Jul 1;12:105. doi: 10.1186/s40900-026-00925-1 (PMC13326187; doi:10.1186/s40900-026-00925-1)
Supplement: Supplementary file 4 — Supplementary Material 4 [file 40900_2026_925_MOESM4_ESM.docx]

**Supplementary file 4. Interview and focus group guides**

**Individual interviews with PPI group members**

**1. General experience of being involved in oncology research**

*Briefly explain how you are/have been involved in research projects (Which organizations? What kind of activities? How often?)*

*Would you describe your involvement in these initiatives as meaningful to you? Does it add value to you as a person? Why/why not?*

**2. Individual added value for PPI contributors**

*What makes initiatives for involving patients in oncology research meaningful? And how do we achieve this? Are there certain challenges that prevent meaningful engagement?*

- ***General support and guidance from professionals***

*What kind of support and guidance from professionals – if any – do you need to be meaningfully involved in research projects? Is that enough now? If not, how can this be improved?*

- ***Education***

*Do patients need training to be involved in research projects in a meaningful way? In which case do patients need additional training? How should training ideally be organized?*

- ***Feedback***

*Is feedback important for patients to be involved in research projects in a meaningful way? Who should provide feedback and how should it be provided?*

- ***Appreciation by professionals***

*Do you feel valued by professionals when you are involved in research projects? Does this make your involvement feel more meaningful?*

***3. Collective added value***

*What added value does PPI bring to cancer patients in general? Do you think that PPI groups currently provide sufficient added value for cancer patients in general? If so, how? If not, how can this be further improved in the future?*

*Based on everything we've discussed today, what do you consider to be the ultimate end goal of PPI in oncology research? What should we collectively – patients, professionals and researchers – achieve with these initiatives?*

**Focus group with PPI coordinators**

**1. General experience with PPI in oncology research**

***Roundtable discussion:*** *Briefly explain how you are involved in PPI? (Which organizations? What kind of activities?)*

***Roundtable discussion:*** *Would you describe your involvement in these initiatives as valuable to yourself? Does it add value to you on a personal or professional level (or both?) Why/why not? What do you learn from the members (patients)? How (and to what extent) is their feedback valuable to you on a personal or professional level?*

**2. Individual added value**

***Group discussion:*** *What added value can PPI bring to group members – participating (former) patients (or patient representatives) – and to cancer patients in general?*

***Group discussion:*** *For which type of research projects/studies could PPI be most valuable?*

***Group discussion:*** *In which study phases could PPI be most valuable?*

***Group discussion:*** *Are current initiatives already delivering enough added value today?*

- *If so, what makes them add value? Are there any improvements that can be made?*
- *If not, how can we ensure that they add more value in the future? (Are there certain challenges that prevent meaningful initiatives and how can we overcome them?)*
- ***General support and guidance (to patients and professionals)***

*What kind of support and guidance (e.g. training) do you offer – if applicable – to patients involved in research projects?*

*Is that enough now? If not, how can that be improved?*

*What about lower educated group members? Do they need more targeted support?*

*And vice versa, do you need extra support to organize patient participation in a meaningful way? Who is responsible for that support (e.g. own organizations, policy makers, research funders, …).*

- ***Budget***

*How do you evaluate the budget for PPI groups in your organizations? Should additional budget be allocated to researchers/research institutions to implement meaningful PPI?*

- **Feedback**

*How do you (or researchers who submit projects) give feedback to the group members? Is feedback important for members to make a meaningful contribution?*

- **Diversity and inclusion**

*Patient participation initiatives mainly attract highly educated members. Do you see this as a problem (too little diversity) for meaningful PPI or even as an opportunity (more available expertise and less training needed)?*

**3. Impact and end goal of patient participation**

***Group discussion:*** *Based on everything we discussed today, what do you consider to be the ultimate end goal of PPI in oncology research? What should we achieve as a collective – patients, professionals and researchers – with these initiatives?*
