# Supplementary material for: What added value does Patient and Public Involvement (PPI) in oncology research bring to cancer patients and what are the challenges in realizing it? A mixed-methods cross-sectional study in four PPI groups in Flanders (Belgium)
Source: Res Involv Engagem. 2026 Jul 1;12:105. doi: 10.1186/s40900-026-00925-1 (PMC13326187; doi:10.1186/s40900-026-00925-1)
Supplement: Supplementary file 5 — Supplementary Material 5 [file 40900_2026_925_MOESM5_ESM.docx]

**Supplementary file 5. Coding trees**

**1. Individual added value of PPI groups**

**Individual added value of PPI groups**

Sense of usefulness

Sense of belonging

Role model

Post professional life

Peer support

Group feeling

Cognition

Knowledge acquisition

Knowledge

**2. Collective added value of PPI groups**

**Collective added value of PPI groups**

Direction

Feasibility

Acceptability

Dissemination

Idea formulation

Education and training

Research types

Patient facing documents

Participant burden

Study recruitment

Patient voice

Patient needs

Research relevance

Patient organizations

Patient networking

**3. Challenges to added value creation**

**Challenges to added value creation**

Training

Diversity & inclusion

Feedback procedures

Support

Additional training

Experiential knowledge

Education level

Health literacy

Cultural diversity

Variability

Role of coordinators

Invisibility

Coordinator support

Funding

Umbrella organization
